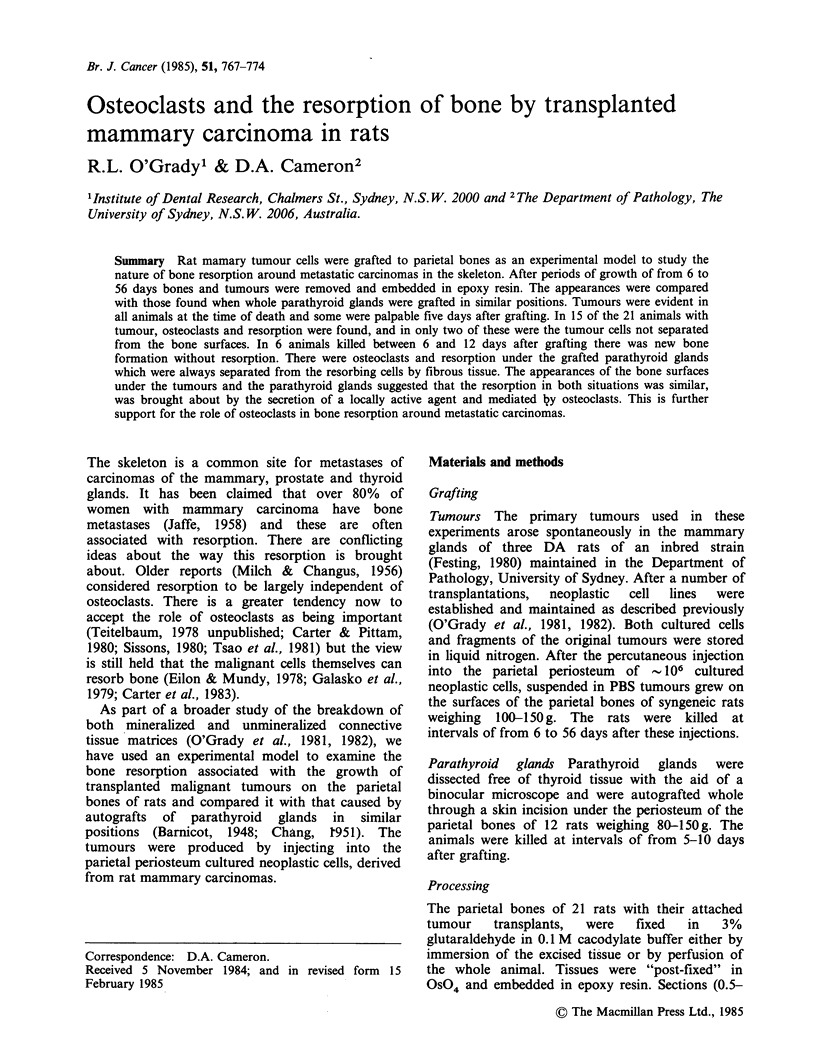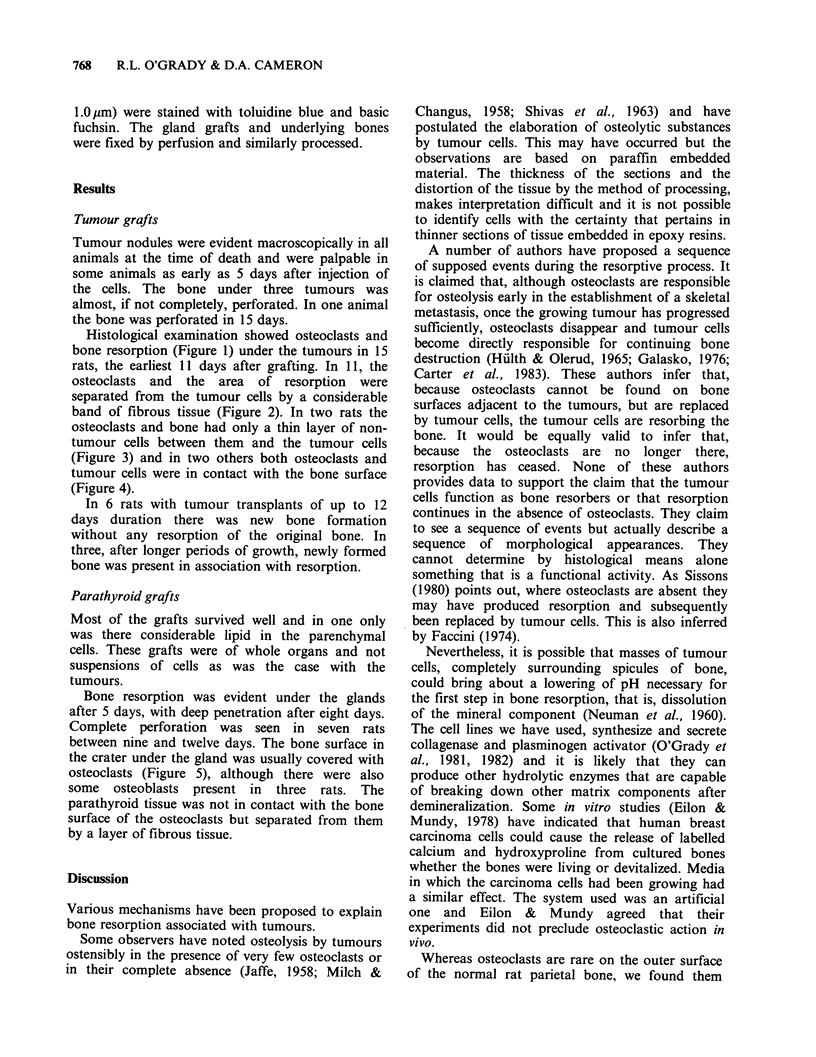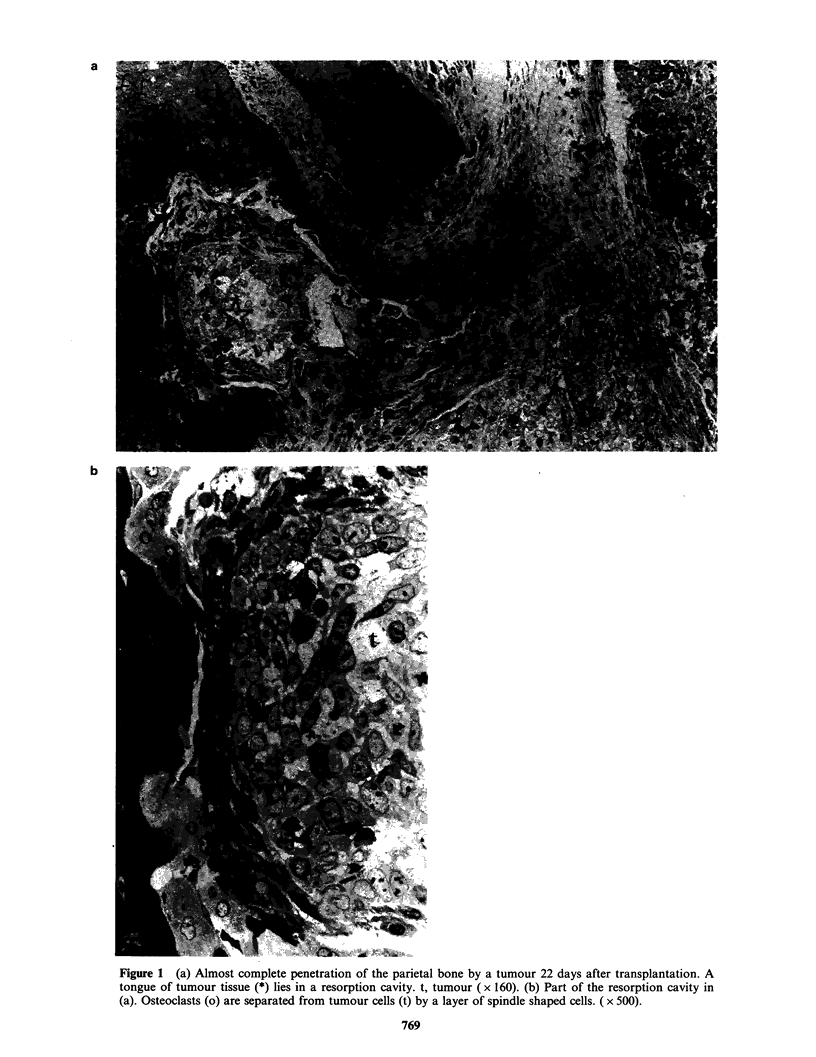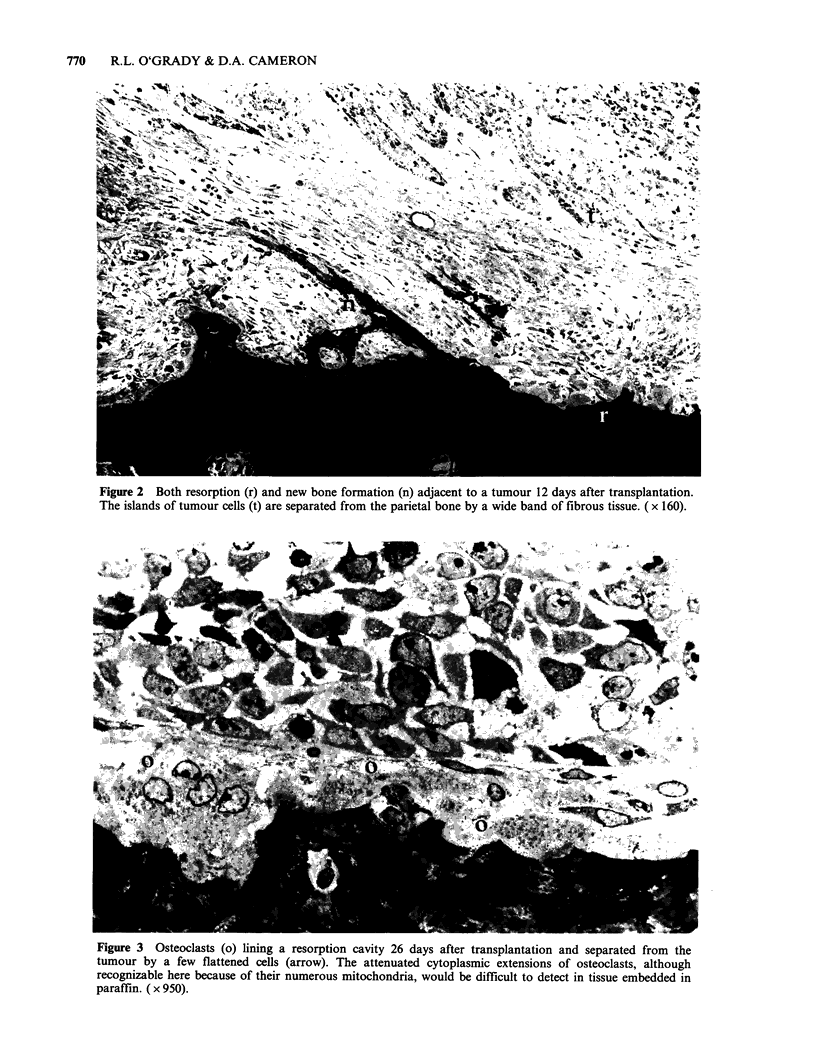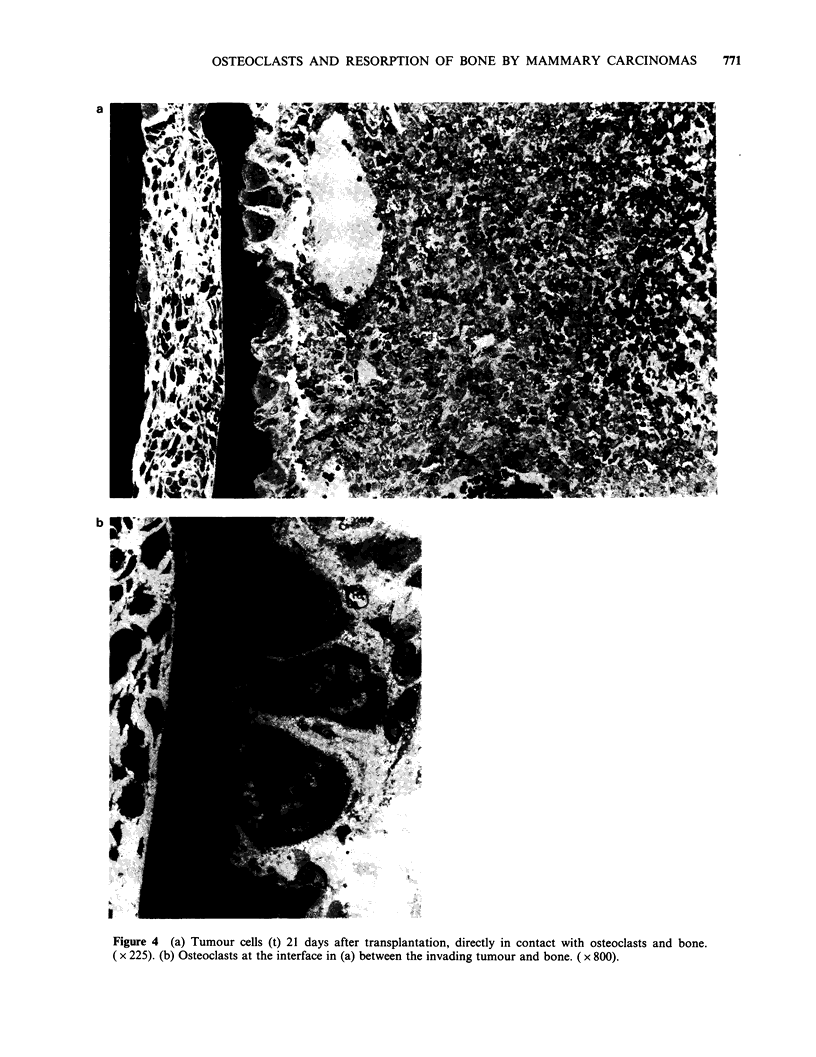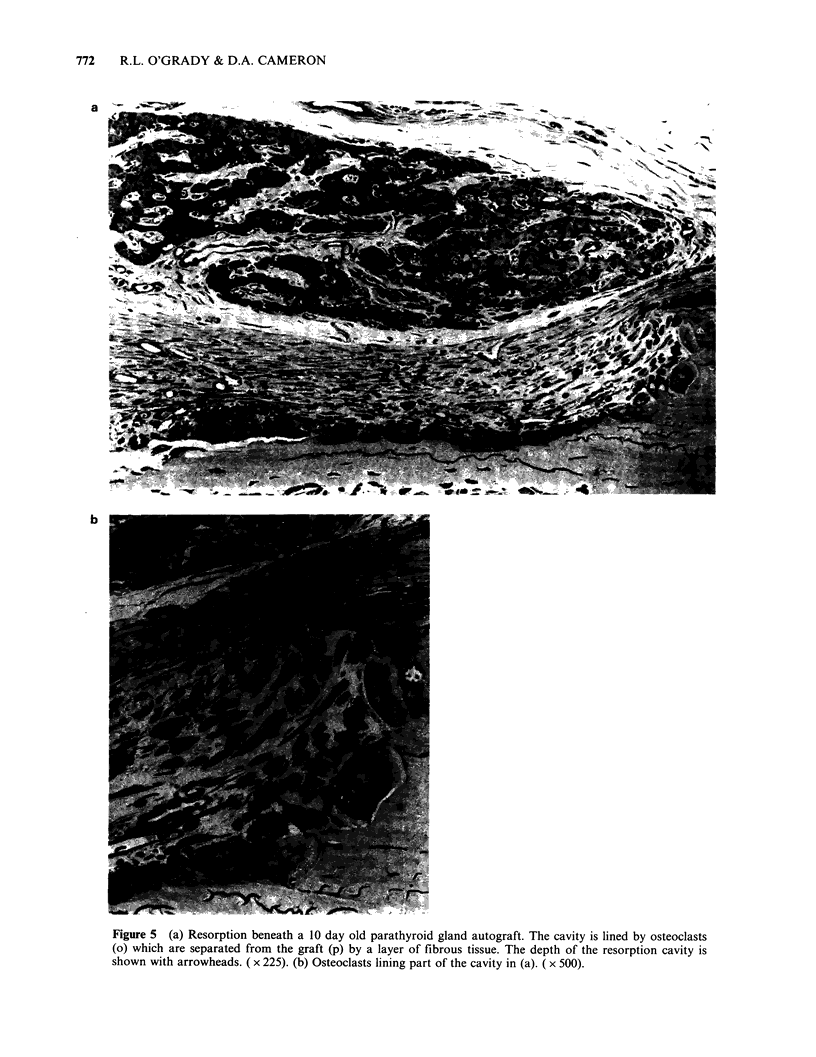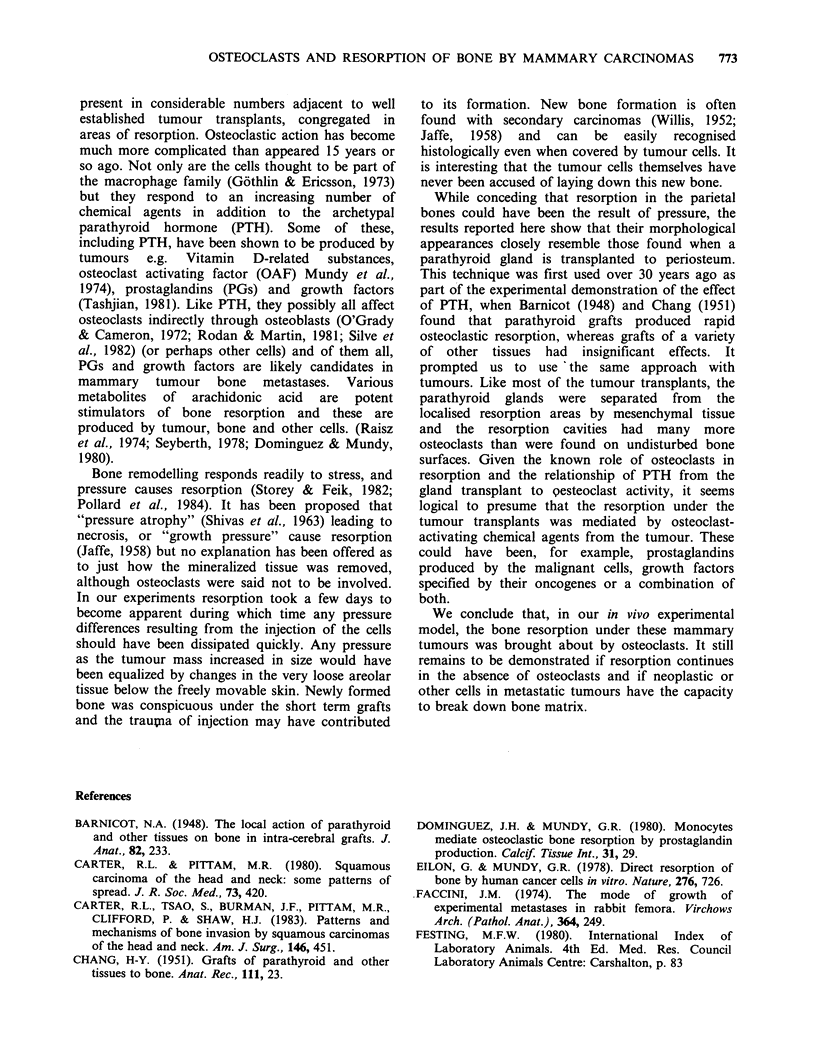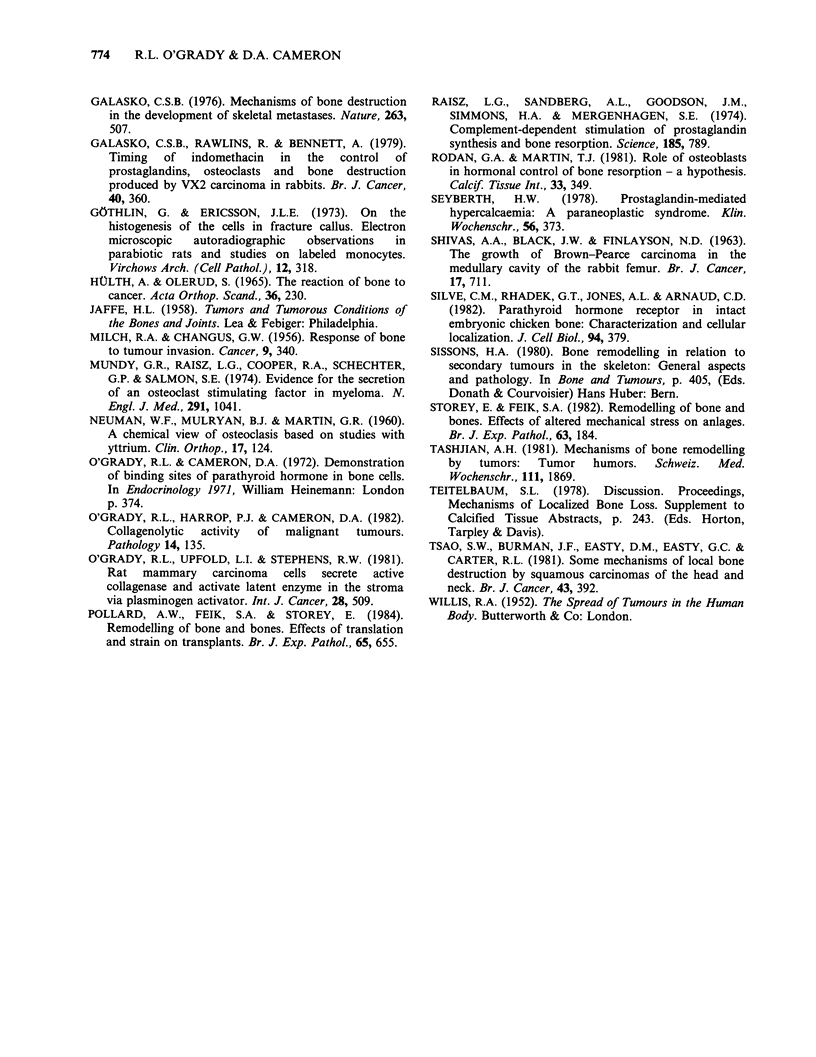# Osteoclasts and the resorption of bone by transplanted mammary carcinoma in rats.

**DOI:** 10.1038/bjc.1985.120

**Published:** 1985-06

**Authors:** R. L. O'Grady, D. A. Cameron

## Abstract

**Images:**


*Br. J. Cancer* (1985), **51**, 767–774

### Osteoclasts and the resorption of bone by transplanted mammary carcinoma in rats

R.L. O'Grady[1] & D.A. Cameron[2]

[1]*Institute of Dental Research, Chalmers St., Sydney, N.S.W. 2000 and* [2]*The Department of Pathology, The University of Sydney, N.S.W. 2006, Australia.*

**Summary** Rat mamary tumour cells were grafted to parietal bones as an experimental model to study the nature of bone resorption around metastatic carcinomas in the skeleton. After periods of growth of from 6 to 56 days bones and tumours were removed and embedded in epoxy resin. The appearances were compared with those found when whole parathyroid glands were grafted in similar positions. Tumours were evident in all animals at the time of death and some were palpable five days after grafting. In 15 of the 21 animals with tumour, osteoclasts and resorption were found, and in only two of these were the tumour cells not separated from the bone surfaces. In 6 animals killed between 6 and 12 days after grafting there was new bone formation without resorption. There were osteoclasts and resorption under the grafted parathyroid glands which were always separated from the resorbing cells by fibrous tissue. The appearances of the bone surfaces under the tumours and the parathyroid glands suggested that the resorption in both situations was similar, was brought about by the secretion of a locally active agent and mediated by osteoclasts. This is further support for the role of osteoclasts in bone resorption around metastatic carcinomas.

The skeleton is a common site for metastases of carcinomas of the mammary, prostate and thyroid glands. It has been claimed that over 80% of women with mammary carcinoma have bone metastases (Jaffe, 1958) and these are often associated with resorption. There are conflicting ideas about the way this resorption is brought about. Older reports (Milch & Changus, 1956) considered resorption to be largely independent of osteoclasts. There is a greater tendency now to accept the role of osteoclasts as being important (Teitelbaum, 1978 unpublished; Carter & Pittam, 1980; Sissons, 1980; Tsao *et al.*, 1981) but the view is still held that the malignant cells themselves can resorb bone (Eilon & Mundy, 1978; Galasko *et al.*, 1979; Carter *et al.*, 1983).

As part of a broader study of the breakdown of both mineralized and unmineralized connective tissue matrices (O'Grady *et al.*, 1981, 1982), we have used an experimental model to examine the bone resorption associated with the growth of transplanted malignant tumours on the parietal bones of rats and compared it with that caused by autografts of parathyroid glands in similar positions (Barnicot, 1948; Chang, 1951). The tumours were produced by injecting into the parietal periosteum cultured neoplastic cells, derived from rat mammary carcinomas.

Correspondence: D.A. Cameron.
Received 5 November 1984; and in revised form 15 February 1985

### Materials and methods

### Grafting

*Tumours* The primary tumours used in these experiments arose spontaneously in the mammary glands of three DA rats of an inbred strain (Festing, 1980) maintained in the Department of Pathology, University of Sydney. After a number of transplantations, neoplastic cell lines were established and maintained as described previously (O'Grady *et al.*, 1981, 1982). Both cultured cells and fragments of the original tumours were stored in liquid nitrogen. After the percutaneous injection into the parietal periosteum of $\sim 10^6$ cultured neoplastic cells, suspended in PBS tumours grew on the surfaces of the parietal bones of syngeneic rats weighing 100–150 g. The rats were killed at intervals of from 6 to 56 days after these injections.

*Parathyroid glands* Parathyroid glands were dissected free of thyroid tissue with the aid of a binocular microscope and were autografted whole through a skin incision under the periosteum of the parietal bones of 12 rats weighing 80–150 g. The animals were killed at intervals of from 5–10 days after grafting.

### Processing

The parietal bones of 21 rats with their attached tumour transplants, were fixed in 3% glutaraldehyde in 0.1 M cacodylate buffer either by immersion of the excised tissue or by perfusion of the whole animal. Tissues were "post-fixed" in $OsO_4$ and embedded in epoxy resin. Sections (0.5–

© The Macmillan Press Ltd., 1985

1.0 $\mu$m) were stained with toluidine blue and basic fuchsin. The gland grafts and underlying bones were fixed by perfusion and similarly processed.

### Results

### *Tumour grafts*

Tumour nodules were evident macroscopically in all animals at the time of death and were palpable in some animals as early as 5 days after injection of the cells. The bone under three tumours was almost, if not completely, perforated. In one animal the bone was perforated in 15 days.

Histological examination showed osteoclasts and bone resorption (Figure 1) under the tumours in 15 rats, the earliest 11 days after grafting. In 11, the osteoclasts and the area of resorption were separated from the tumour cells by a considerable band of fibrous tissue (Figure 2). In two rats the osteoclasts and bone had only a thin layer of non-tumour cells between them and the tumour cells (Figure 3) and in two others both osteoclasts and tumour cells were in contact with the bone surface (Figure 4).

In 6 rats with tumour transplants of up to 12 days duration there was new bone formation without any resorption of the original bone. In three, after longer periods of growth, newly formed bone was present in association with resorption.

### *Parathyroid grafts*

Most of the grafts survived well and in one only was there considerable lipid in the parenchymal cells. These grafts were of whole organs and not suspensions of cells as was the case with the tumours.

Bone resorption was evident under the glands after 5 days, with deep penetration after eight days. Complete perforation was seen in seven rats between nine and twelve days. The bone surface in the crater under the gland was usually covered with osteoclasts (Figure 5), although there were also some osteoblasts present in three rats. The parathyroid tissue was not in contact with the bone surface of the osteoclasts but separated from them by a layer of fibrous tissue.

### Discussion

Various mechanisms have been proposed to explain bone resorption associated with tumours.

Some observers have noted osteolysis by tumours ostensibly in the presence of very few osteoclasts or in their complete absence (Jaffe, 1958; Milch & Changus, 1958; Shivas *et al.,* 1963) and have postulated the elaboration of osteolytic substances by tumour cells. This may have occurred but the observations are based on paraffin embedded material. The thickness of the sections and the distortion of the tissue by the method of processing, makes interpretation difficult and it is not possible to identify cells with the certainty that pertains in thinner sections of tissue embedded in epoxy resins.

A number of authors have proposed a sequence of supposed events during the resorptive process. It is claimed that, although osteoclasts are responsible for osteolysis early in the establishment of a skeletal metastasis, once the growing tumour has progressed sufficiently, osteoclasts disappear and tumour cells become directly responsible for continuing bone destruction (Hülth & Olerud, 1965; Galasko, 1976; Carter *et al.,* 1983). These authors infer that, because osteoclasts cannot be found on bone surfaces adjacent to the tumours, but are replaced by tumour cells, the tumour cells are resorbing the bone. It would be equally valid to infer that, because the osteoclasts are no longer there, resorption has ceased. None of these authors provides data to support the claim that the tumour cells function as bone resorbers or that resorption continues in the absence of osteoclasts. They claim to see a sequence of events but actually describe a sequence of morphological appearances. They cannot determine by histological means alone something that is a functional activity. As Sissons (1980) points out, where osteoclasts are absent they may have produced resorption and subsequently been replaced by tumour cells. This is also inferred by Faccini (1974).

Nevertheless, it is possible that masses of tumour cells, completely surrounding spicules of bone, could bring about a lowering of pH necessary for the first step in bone resorption, that is, dissolution of the mineral component (Neuman *et al.,* 1960). The cell lines we have used, synthesize and secrete collagenase and plasminogen activator (O'Grady *et al.,* 1981, 1982) and it is likely that they can produce other hydrolytic enzymes that are capable of breaking down other matrix components after demineralization. Some *in vitro* studies (Eilon & Mundy, 1978) have indicated that human breast carcinoma cells could cause the release of labelled calcium and hydroxyproline from cultured bones whether the bones were living or devitalized. Media in which the carcinoma cells had been growing had a similar effect. The system used was an artificial one and Eilon & Mundy agreed that their experiments did not preclude osteoclastic action *in vivo.*

Whereas osteoclasts are rare on the outer surface of the normal rat parietal bone, we found them

**Figure 1** (a) Almost complete penetration of the parietal bone by a tumour 22 days after transplantation. A tongue of tumour tissue (*) lies in a resorption cavity. t, tumour ( × 160). (b) Part of the resorption cavity in (a). Osteoclasts (o) are separated from tumour cells (t) by a layer of spindle shaped cells. ( × 500).

**Figure 2** Both resorption (r) and new bone formation (n) adjacent to a tumour 12 days after transplantation. The islands of tumour cells (t) are separated from the parietal bone by a wide band of fibrous tissue. (×160).

**Figure 3** Osteoclasts (o) lining a resorption cavity 26 days after transplantation and separated from the tumour by a few flattened cells (arrow). The attenuated cytoplasmic extensions of osteoclasts, although recognizable here because of their numerous mitochondria, would be difficult to detect in tissue embedded in paraffin. (×950).

**Figure 4** (a) Tumour cells (t) 21 days after transplantation, directly in contact with osteoclasts and bone. ( × 225). (b) Osteoclasts at the interface in (a) between the invading tumour and bone. ( × 800).

**Figure 5** (a) Resorption beneath a 10 day old parathyroid gland autograft. The cavity is lined by osteoclasts (o) which are separated from the graft (p) by a layer of fibrous tissue. The depth of the resorption cavity is shown with arrowheads. ($\times 225$). (b) Osteoclasts lining part of the cavity in (a). ($\times 500$).

present in considerable numbers adjacent to well established tumour transplants, congregated in areas of resorption. Osteoclastic action has become much more complicated than appeared 15 years or so ago. Not only are the cells thought to be part of the macrophage family (Göthlin & Ericsson, 1973) but they respond to an increasing number of chemical agents in addition to the archetypal parathyroid hormone (PTH). Some of these, including PTH, have been shown to be produced by tumours e.g. Vitamin D-related substances, osteoclast activating factor (OAF) Mundy *et al.*, 1974), prostaglandins (PGs) and growth factors (Tashjian, 1981). Like PTH, they possibly all affect osteoclasts indirectly through osteoblasts (O'Grady & Cameron, 1972; Rodan & Martin, 1981; Silve *et al.*, 1982) (or perhaps other cells) and of them all, PGs and growth factors are likely candidates in mammary tumour bone metastases. Various metabolites of arachidonic acid are potent stimulators of bone resorption and these are produced by tumour, bone and other cells. (Raisz *et al.*, 1974; Seyberth, 1978; Dominguez & Mundy, 1980).

Bone remodelling responds readily to stress, and pressure causes resorption (Storey & Feik, 1982; Pollard *et al.*, 1984). It has been proposed that "pressure atrophy" (Shivas *et al.*, 1963) leading to necrosis, or "growth pressure" cause resorption (Jaffe, 1958) but no explanation has been offered as to just how the mineralized tissue was removed, although osteoclasts were said not to be involved. In our experiments resorption took a few days to become apparent during which time any pressure differences resulting from the injection of the cells should have been dissipated quickly. Any pressure as the tumour mass increased in size would have been equalized by changes in the very loose areolar tissue below the freely movable skin. Newly formed bone was conspicuous under the short term grafts and the trauma of injection may have contributed to its formation. New bone formation is often found with secondary carcinomas (Willis, 1952; Jaffe, 1958) and can be easily recognised histologically even when covered by tumour cells. It is interesting that the tumour cells themselves have never been accused of laying down this new bone.

While conceding that resorption in the parietal bones could have been the result of pressure, the results reported here show that their morphological appearances closely resemble those found when a parathyroid gland is transplanted to periosteum. This technique was first used over 30 years ago as part of the experimental demonstration of the effect of PTH, when Barnicot (1948) and Chang (1951) found that parathyroid grafts produced rapid osteoclastic resorption, whereas grafts of a variety of other tissues had insignificant effects. It prompted us to use the same approach with tumours. Like most of the tumour transplants, the parathyroid glands were separated from the localised resorption areas by mesenchymal tissue and the resorption cavities had many more osteoclasts than were found on undisturbed bone surfaces. Given the known role of osteoclasts in resorption and the relationship of PTH from the gland transplant to osteoclast activity, it seems logical to presume that the resorption under the tumour transplants was mediated by osteoclast-activating chemical agents from the tumour. These could have been, for example, prostaglandins produced by the malignant cells, growth factors specified by their oncogenes or a combination of both.

We conclude that, in our *in vivo* experimental model, the bone resorption under these mammary tumours was brought about by osteoclasts. It still remains to be demonstrated if resorption continues in the absence of osteoclasts and if neoplastic or other cells in metastatic tumours have the capacity to break down bone matrix.

### References

BARNICOT, N.A. (1948). The local action of parathyroid and other tissues on bone in intra-cerebral grafts. *J. Anat.*, **82,** 233.

CARTER, R.L. & PITTAM, M.R. (1980). Squamous carcinoma of the head and neck: some patterns of spread. *J. R. Soc. Med.*, **73,** 420.

CARTER, R.L., TSAO, S., BURMAN, J.F., PITTAM, M.R., CLIFFORD, P. & SHAW, H.J. (1983). Patterns and mechanisms of bone invasion by squamous carcinomas of the head and neck. *Am. J. Surg.*, **146,** 451.

CHANG, H-Y. (1951). Grafts of parathyroid and other tissues to bone. *Anat. Rec.*, **111,** 23.

DOMINGUEZ, J.H. & MUNDY, G.R. (1980). Monocytes mediate osteoclastic bone resorption by prostaglandin production. *Calcif. Tissue Int.*, **31,** 29.

EILON, G. & MUNDY, G.R. (1978). Direct resorption of bone by human cancer cells *in vitro*. *Nature*, **276,** 726.

FACCINI, J.M. (1974). The mode of growth of experimental metastases in rabbit femora. *Virchows Arch. (Pathol. Anat.)*, **364,** 249.

FESTING, M.F.W. (1980). International Index of Laboratory Animals. 4th Ed. Med. Res. Council Laboratory Animals Centre: Carshalton, p. 83

GALASKO, C.S.B. (1976). Mechanisms of bone destruction in the development of skeletal metastases. *Nature*, **263**, 507.

GALASKO, C.S.B., RAWLINS, R. & BENNETT, A. (1979). Timing of indomethacin in the control of prostaglandins, osteoclasts and bone destruction produced by VX2 carcinoma in rabbits. *Br. J. Cancer*, **40**, 360.

GÖTHLIN, G. & ERICSSON, J.L.E. (1973). On the histogenesis of the cells in fracture callus. Electron microscopic autoradiographic observations in parabiotic rats and studies on labeled monocytes. *Virchows Arch. (Cell Pathol.)*, **12**, 318.

HÜLTH, A. & OLERUD, S. (1965). The reaction of bone to cancer. *Acta Orthop. Scand.*, **36**, 230.

JAFFE, H.L. (1958). *Tumors and Tumorous Conditions of the Bones and Joints*. Lea & Febiger: Philadelphia.

MILCH, R.A. & CHANGUS, G.W. (1956). Response of bone to tumour invasion. *Cancer*, **9**, 340.

MUNDY, G.R., RAISZ, L.G., COOPER, R.A., SCHECHTER, G.P. & SALMON, S.E. (1974). Evidence for the secretion of an osteoclast stimulating factor in myeloma. *N. Engl. J. Med.*, **291**, 1041.

NEUMAN, W.F., MULRYAN, B.J. & MARTIN, G.R. (1960). A chemical view of osteoclasis based on studies with yttrium. *Clin. Orthop.*, **17**, 124.

O'GRADY, R.L. & CAMERON, D.A. (1972). Demonstration of binding sites of parathyroid hormone in bone cells. In *Endocrinology 1971*, William Heinemann: London p. 374.

O'GRADY, R.L., HARROP, P.J. & CAMERON, D.A. (1982). Collagenolytic activity of malignant tumours. *Pathology* **14**, 135.

O'GRADY, R.L., UPFOLD, L.I. & STEPHENS, R.W. (1981). Rat mammary carcinoma cells secrete active collagenase and activate latent enzyme in the stroma via plasminogen activator. *Int. J. Cancer*, **28**, 509.

POLLARD, A.W., FEIK, S.A. & STOREY, E. (1984). Remodelling of bone and bones. Effects of translation and strain on transplants. *Br. J. Exp. Pathol.*, **65**, 655.

RAISZ, L.G., SANDBERG, A.L., GOODSON, J.M., SIMMONS, H.A. & MERGENHAGEN, S.E. (1974). Complement-dependent stimulation of prostaglandin synthesis and bone resorption. *Science*, **185**, 789.

RODAN, G.A. & MARTIN, T.J. (1981). Role of osteoblasts in hormonal control of bone resorption – a hypothesis. *Calcif. Tissue Int.*, **33**, 349.

SEYBERTH, H.W. (1978). Prostaglandin-mediated hypercalcaemia: A paraneoplastic syndrome. *Klin. Wochenschr.*, **56**, 373.

SHIVAS, A.A., BLACK, J.W. & FINLAYSON, N.D. (1963). The growth of Brown–Pearce carcinoma in the medullary cavity of the rabbit femur. *Br. J. Cancer*, **17**, 711.

SILVE, C.M., RHADEK, G.T., JONES, A.L. & ARNAUD, C.D. (1982). Parathyroid hormone receptor in intact embryonic chicken bone: Characterization and cellular localization. *J. Cell Biol.*, **94**, 379.

SISSONS, H.A. (1980). Bone remodelling in relation to secondary tumours in the skeleton: General aspects and pathology. In *Bone and Tumours*, p. 405, (Eds. Donath & Courvoisier) Hans Huber: Bern.

STOREY, E. & FEIK, S.A. (1982). Remodelling of bone and bones. Effects of altered mechanical stress on anlages. *Br. J. Exp. Pathol.*, **63**, 184.

TASHJIAN, A.H. (1981). Mechanisms of bone remodelling by tumors: Tumor humors. *Schweiz. Med. Wochenschr.*, **111**, 1869.

TEITELBAUM, S.L. (1978). Discussion. Proceedings, Mechanisms of Localized Bone Loss. Supplement to Calcified Tissue Abstracts, p. 243. (Eds. Horton, Tarpley & Davis).

TSAO, S.W., BURMAN, J.F., EASTY, D.M., EASTY, G.C. & CARTER, R.L. (1981). Some mechanisms of local bone destruction by squamous carcinomas of the head and neck. *Br. J. Cancer*, **43**, 392.

WILLIS, R.A. (1952). *The Spread of Tumours in the Human Body*. Butterworth & Co: London.